# The impact of lockdown on pediatric ED visits and hospital admissions during the COVID19 pandemic: a multicenter analysis and review of the literature

**DOI:** 10.1007/s00431-021-04015-0

**Published:** 2021-03-15

**Authors:** Matthijs D. Kruizinga, Daphne Peeters, Mirjam van Veen, Marlies van Houten, Jantien Wieringa, Jeroen G. Noordzij, Jolita Bekhof, Gerdien Tramper-Stranders, Nienke J. Vet, Gertjan J. A. Driessen

**Affiliations:** 1 Juliana Children’s Hospital (Haga Teaching hospital), Els Borst-Eilersplein 275, 2545 AA The Hague, the Netherlands; 2 Centre for Human Drug Research, Leiden, the Netherlands; 3 Department of Pediatrics, Groene Hart Ziekenhuis, Gouda, the Netherlands; 4 Department of Pediatrics, Spaarne Gasthuis, Hoofddorp/Haarlem, the Netherlands; 5 Department of Pediatrics, Haaglanden Medical Centre, The Hague, the Netherlands; 6 Department of Pediatrics, Reinier de Graaf Ziekenhuis, Delft, the Netherlands; 7 Department of Pediatrics, Isala, Zwolle, the Netherlands; 8 Department of Pediatrics, Franciscus Gasthuis& Vlietland, Rotterdam, the Netherlands; 9 Department of Pediatrics, St Antonius Ziekenhuis, Nieuwegein, the Netherlands; 10 Department of Pediatrics, Maastricht University Medical Center, Maastricht, the Netherlands

**Keywords:** COVID-19, Corona, SARS-CoV-2, Lockdown, Pediatrics, Admissions, ED visits

## Abstract

The coronavirus disease 2019 pandemic has enormous impact on society and healthcare. Countries imposed lockdowns, which were followed by a reduction in care utilization. The aims of this study were to quantify the effects of lockdown on pediatric care in the Netherlands, to elucidate the cause of the observed reduction in pediatric emergency department (ED) visits and hospital admissions, and to summarize the literature regarding the effects of lockdown on pediatric care worldwide. ED visits and hospital admission data of 8 general hospitals in the Netherlands between January 2016 and June 2020 were summarized per diagnosis group (communicable infections, noncommunicable infections, (probable) infection-related, and noninfectious). The effects of lockdown were quantified with a linear mixed effects model. A literature review regarding the effect of lockdowns on pediatric clinical care was performed. In total, 126,198 ED visits and 47,648 admissions were registered in the study period. The estimated reduction in general pediatric care was 59% and 56% for ED visits and admissions, respectively. The largest reduction was observed for communicable infections (ED visits: 76%; admissions: 77%), whereas the reduction in noninfectious diagnoses was smaller (ED visits 36%; admissions: 37%). Similar reductions were reported worldwide, with decreases of 30–89% for ED visits and 19–73% for admissions.

*Conclusion*: Pediatric ED utilization and hospitalization during lockdown were decreased in the Netherlands and other countries, which can largely be attributed to a decrease in communicable infectious diseases. Care utilization for other conditions was decreased as well, which may indicate that care avoidance during a pandemic is significant.

**Table:** 

**What is Known:** *• The COVID-19 pandemic had enormous impact on society.* *• Countries imposed lockdowns to curb transmission rates, which were followed by a reduction in care utilization worldwide.*	
**What is New**: *• The Dutch lockdown caused a significant decrease in pediatric ED utilization and hospitalization, especially in ED visits and hospital admissions because of infections that were not caused by SARS-CoV-2.* *• Care utilization for noninfectious diagnoses was decreased as well, which may indicate that pediatric care avoidance during a pandemic is significant.*

**Supplementary Information:**

The online version contains supplementary material available at 10.1007/s00431-021-04015-0.

## Introduction

In 2020, the world was shocked by the pandemic of coronavirus disease 2019 (COVID-19) caused by the SARS-CoV-2 virus [1]. During the first 4 months of the year, transmission rates in Europe were of such a significant magnitude that several governments were forced to impose harsh lockdowns [2]. In the Netherlands, the first COVID-19 case was diagnosed on the 27th of February, and the government imposed a limited lockdown on the 23rd of March due to spiraling infection numbers. This lockdown led to closure of schools, daycares, and catering industry until the 11th of May and the 1st of June, respectively [3].

Lockdowns are serious interventions and are not expected to only have a specific effect on COVID-19 transmission but on all transmissible infectious diseases. This has been reported in similar situations in the past [4]. This effect may be especially pronounced in the field of pediatrics, as 28% of diagnoses in pediatric emergency departments (EDs) are attributed to infectious disease [5]. As expected, a decrease in the total numbers of pediatric admissions and visits to the emergency department (ED) has followed lockdowns worldwide [6–8]. However, it is unclear whether this reduction is solely due to a decrease in transmissible infections or also by behavioral changes around healthcare utilization. For example, there have been numerous reported examples of avoidance of care due to fear of a hospital environment, which is potentially disastrous [9–11].

Although the overall reduction in pediatric patients seeking care has been widely reported, stratification of specific disease groups has not been performed. If avoidance of care is a significant factor, one would expect a similar reduction in admissions and ED visits due to noninfectious disease compared to visits for transmissible infectious disease. Furthermore, lockdown as intervention allows for a unique opportunity to investigate the incidence of diagnoses that are assumed, but not definitively proven, to be related to or luxated by transmissible infections.

The aim of this study was to quantify the impact of the Dutch lockdown on pediatric clinical care in the Netherlands, to assess whether the impact can be attributed solely to a decrease in transmissible infections, and to critically review and summarize the international literature regarding the effect of lockdown on pediatric clinical care.

## Methods

### Study design and ethics

This was a retrospective multicenter study including 8 general hospitals in the Netherlands, mainly located in urban settings. None of the participating centers harbors a PICU. The study protocol was submitted to the medical ethics committee LDD (Leiden, the Netherlands), who judged that the protocol did not fall under the purview of the Dutch Law for Research with Human Subjects (WMO). The study was conducted in line with the general data protection regulation (GDPR).

### Data collection and aggregation

Raw ED visit and admission data for each month between the 1st of January 2016 and the 30th of June 2020 were exported from the electronic patient records of each participating hospital. A filter was applied to exclude all subjects older than 18 years, admissions, ED visits on the surgery, or orthopedic service and admissions due to standard neonatal care. After merging the 8 datasets, a list of unique diagnoses was exported, and each individual diagnosis was allocated to one of the following 4 groups: communicable infections such as respiratory tract infections; infection-related, e.g., reactive arthritis; noncommunicable infections such as urinary tract infections; and noninfectious, for instance, failure to thrive. A list of the included diagnoses in each category can be found in Supplementary Table S1.

### Statistics

The sum of ED visits and admissions per center for each individual month was calculated. Because of inter-center difference, data was normalized. The mean number of ED visits each month per center in 2016–2019 was calculated and fixed at 100%, after which the relative percentage of admissions and ED visits during each month in 2016–2020 was calculated. A mixed effects model with month and year (2016–2019 vs 2020) as fixed effect and study center as random intercept was used to describe the relative reduction and 95% confidence interval (CI) in admissions and ED visits in 2020 for all diagnoses and for each diagnostic category separately. Bonferroni’s method was used to adjust for multiple comparisons. Previous studies mention a substantial proportion of preventable ED visits in children which are due to non-urgent or trivial issues [12]. We therefore calculated the admission/ED ratio in order to estimate the acuity of ED visits. R-version 3.6.2 with the lme4 and ggeffects packages was used for statistical analysis.

### Literature review

EMBASE, Medline Ovid, Web of Science Core Collection, and CINAHL EBSCOhost databases were searched for articles describing the effect of lockdown on pediatric clinic care (Supplementary Text S2). All study and publication types were included if they were published between January 1, 2020, and October 15, 2020 (date of the search), and contained any quantitative information on ED visits or hospitalization of children (0–18 years) during the COVID-19 pandemic. Studies that only described patient groups with specific diagnoses and/or COVID-19 or adult patients were excluded. Also, articles were excluded if the full text was not available or if it was written in another language than English. The selected articles were summarized in tables and arranged by country. The average reduction in ED visits per country was correlated with the maximum stringency of the lockdown measures during the first wave of COVID-19 (up until June 2020) [13].

## Results

### General characteristics

In total, 126,198 ED visits and 47,648 admissions were registered in the 8 participating centers between January 2016 and June 2020. During that period, 46% of ED visits and 41% of admissions were due to communicable infections, and 37% of ED visits and 40% of admissions were related to noninfectious disease. In contrast, only 2.8% of ED visits and 2.9% of admissions were associated with noncommunicable infections. Of all patients, 70% were aged younger than 4 years old, 20% were between 4 and 11 years old, and 10% were 12 years or older.

### Effect of lockdown

**Fig. 1:**
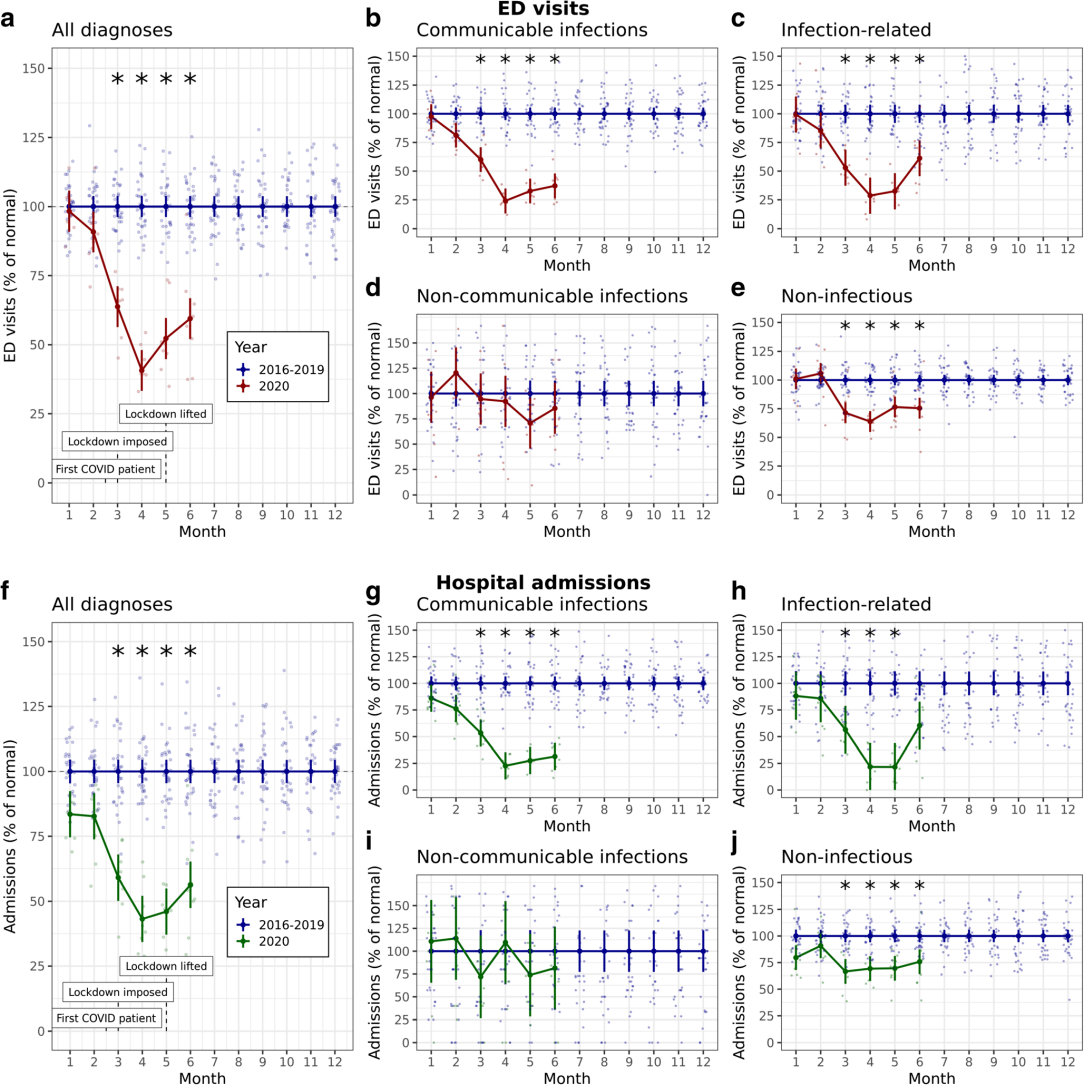
Relative frequency of ED visits and ward admissions. Estimated effect of lockdown on ED visits (**a**–**e**) and pediatric hospital admissions (**f**–**j**). Blue lines indicate the average level during years 2016–2019 (100%). The red line indicates the relative amount of ED visits for each month in 2020. The green line indicates that relative amount of hospital admissions for each month in 2020. Error bars represent the 95% confidence interval of the estimated marginal mean, and each dot is 1 month per hospital. The asterisk (*) indicates a significant difference between 2016 and 2020 (*p* < 0.05, after Bonferroni correction)

The estimated relative differences in ED visits and admissions between 2020 and 2016–2019 are displayed in Fig. 1a and f, respectively. A statistically significant reduction was observed from February 2020 onwards, the month in which the first COVID-19 patients were diagnosed. The largest contrast was observed in April 2020, the month after lockdown was imposed, when the estimated reduction was 59% (95% CI 51–68%, p < 0.001) for ED visits and 57% (95% CI 47–67%, *p* < 0.001) for admissions. Contrary to the number of visits, the mean ratio between admissions and ED visits was not different in 2020 compared to 2016–2019 (Supplementary Figure S3). The similar ratio of patients needing hospitalization suggests that the level of acuity did not change substantially during the national lockdown.

Stratification per disease group (Fig. 1b–e, g–j) showed that the largest reduction in both ED visits and admissions was observed in the communicable infections category. The estimated reduction in April was similar for ED visits (76% (95% CI 64–88%, *p* < 0.001) and admissions (77% (95% CI 63–92%, *p* < 0.001). For diagnoses assumed to be related to infections, an identical reduction was observed, with a reduction of ED visits of 71% (95% CI 54–89%, *p* < 0.001) and reduction in admissions of 78% (95%, CI 53–103%, *p* < 0.001). On the contrary, no significant effect on noncommunicable infections was observed. The largest reduction in noninfectious disease was found in April for ED visits (36% reduction (95% CI 26–46%, *p* < 0.001) and in March for admissions: 33% (95% CI 20–46%, *p* < 0.001). Estimated effects per category for each individual month are displayed in Supplementary Table S4.

Finally, the effect of the lockdown ED visits and admissions related to respiratory infections, diabetes, and mental disease was estimated (Supplementary Figure S5).

### Review of the literature

In total, 1.775 publications were screened for eligibility (Supplementary Figure 6), 33 of which reported the effect of COVID-19 or lockdown on emergency care utilization for general pediatrics. Most publications were from Italy (*n*=12) [7, 14–24] or from the USA (*n*=8) [25–32]. An overview of the range of reported effects for individual countries is shown in Table 1. Three studies compared data of 2020 with the month(s) before lockdown [21, 22, 33], all other studies compared it with previous years. In one study, medical professionals were asked about the magnitude of the effect of COVID-19 on pediatric ED visits [32]. No study reported the four diagnosis categories as described here. Declines in the number of ED visits due to the COVID-19 pandemic were widely reported and ranged from 30 to 89% [6, 34]. In Italy and the USA, a bigger decline in ED visits of children with non-acute illness or low triage codes was reported, causing a larger proportion of ED visits with higher acuity in most countries [14, 17, 20, 28, 35], although one Irish study reported no change after lockdown [36]. The absolute admission count during lockdowns decreased by 19–73% in all studies, except one [17]. The admission/ED visit ratio was reported to show an increase in Italy [14–19], Germany [37], and the USA [26, 28], but not in Ireland [38]. There was no correlation between the reduction in ED visits and the maximum stringency during the study period (*R* = 0.46, *p* = 0.098, Supplementary Figure S7).

**Table 1:** Overview of the effect of the COVID-19 pandemic on ED visits and hospitalization in general pediatrics compared to pre-COVID-19 periods

Country	Reduction of ED visits	Increase in hospital admission/ED ratio	Reduction of hospital admissions	Reference	Maximum stringency of the lockdown during the study period^†^
The Netherlands	59%	Remained stable at 35–45%	56%	Current study	79.63
Italy	62–88%	From 0.5–20 to 0.9–41%	31–71%; 19% increase in one hospital	[7, 14–24]^#^	93.52
Spain	65%	-	-	[35]	85.19
Germany	64%	From 14 to 27%	38%	[37]	76.85
France	68%	-	45%	[40]	87.96
Austria	83%	-	-	[53]	81.48
UK	30–66%*	-	-	[6, 54]	79.63
Ireland	46–54%	Remained stable (14–15%)	41–54%	[36, 38]	90.74
Finland	65–65%	-	45–60%	[33]^#^	67.59
South Africa	58%	-	-	[41]	87.96
Morocco	74%	-	42%	[55]	93.52
USA	48–87%	From 19–20 to 22–24%	73%	[25–32]	72.69
Argentina	89%	-	-	[34]	100
Australia	47%	-	-	[52]	73.15

^*^Isba et al. [6] reported data from March, whereas lockdown was issued only after March 23, 2020

^#^Vierucci et al. [21], Ciofi Degli et al. [22], and Kuitunen et al. [33] compared data with the period before lockdown/behavioral measures, instead of the previous year(s)

^$^Data from Walker et al. [32] was based on a questionnaire among healthcare professionals

^†^Government Response Stringency Index (range 0 to 100, 100 is strictest) [13]

## Discussion

Emergency department visits and hospital admissions were impressively decreased during COVID-19 lockdowns, especially for children with communicable infections. When determining the reason for the observed effects, we expect that three separate caused have played some part in the observed reduction in clinical visits.

First, the closure of school and daycares led to an immediate and dramatic decrease in contacts between children and, as a result, in transmissible infections, which are the cause of a large proportion of pediatric healthcare visits. The current study supports this hypothesis, as the largest observed reduction in ED visits and admissions was found for transmissible infections and infection-related diagnoses. If this reduction could not be attributed to this effect, a reduction of similar size would be expected in the other categories as well. This factor could be considered a positive consequence of a lockdown, which may have led to a decrease in morbidity and healthcare costs. The number of children visiting the ED because of a respiratory illness was reduced more compared to other pediatric diagnoses in other countries as well. Hartnett et al. reported that the largest declines in the number of ED visits were seen in children aged ≤10 years for respiratory symptoms (78%), viral infections (79%), influenza (97%), and otitis (85%) [39]. A large time series analysis in France also reported a 70% reduction for the common cold, otitis media, and gastro-enteritis. In this study, visits because of urinary tract infections (UTIs) were not affected, and UTI admissions even increased by 21%, which is in line with our findings [40]. Furthermore, several Italian hospitals saw a decline of 60–96% for presentations because of fever and respiratory or other infections [18–23]. Other countries reported a decrease in respiratory infections between 52 and 98% as well, and this reduction was generally larger than for other diagnosis groups [8, 33, 36, 37, 41, 42], except for two studies in Spain, a country with a harsh lockdown [13]. Here, the reduction in the number of ED visits because of respiratory symptoms was identical to the overall reduction in visits [16, 35]. In addition to reduced ED visits, our data also showed a decrease in infection-related hospitalizations, which was also observed in Denmark [43].

A second factor of influence could have been the fact that the pandemic led to an extreme demand on the healthcare sector and that clinical care in the hospital largely revolved around COVID-19 patients. Fear of SARS-CoV-2 infection and a desire to not raise demand on the healthcare sector even further may have caused parents to refrain from going to the hospital with their children, despite worsening symptoms [44]. In that case, a reduction in noncommunicable infections and noninfectious diagnoses would be expected. Our data indicates a significant decrease in clinical care related to noninfectious diagnoses, although the reduction was less compared nontransmissible infections. Interestingly, the ratio between admissions and ED visits remained constant in 2020 compared to 2016–2019. If avoidance of care would lead to more children with high acuity illness due to delayed presentation to the ED, this ratio would be expected to rise compared to the previous years. The fact that this was not the case gives reassurance that the avoidance of care did not lead to adverse consequences on a large scale in the Netherlands. However, we did not take disease severity or length of stay into account in our analyses, and more than 50 anecdotal reports regarding collateral harm due to delayed presentation were reported in the Netherlands [45]. However, several countries reported a higher admission/ED visit ratio, and studies that analyzed triage codes found that overall reasons for the ED visit were associated with a higher acuity of disease [28, 41, 46]. Furthermore, there is ample international evidence that point to adverse consequences due to care avoidance. For example, although Dayal et al. reported a 75% reduction in the number of new diabetes cases during lockdown, the authors described three patients who have been diagnosed during lockdown and suffered from severe diabetic keto-acidosis, in which parents declared they delayed seeking care due to COVID-19 circumstances [47].

Third, it is possible that parents and other caregivers experienced less pressure than usual to seek prompt clinical care for their children in the event of illness. During lockdown, working from home was mandatory and childcare was necessarily combined with professional activities. Additionally, reduced external feedback regarding health from schoolteachers, daycare workers, or grandparents could also lead to a reduced extrinsic motivation to visit the ED [48]. Some evidence of this effect could be derived from the current analysis, since numbers of noninfectious diagnoses were also reduced. However, it is not possible to completely isolate this effect from the avoidance of care that is assumed to be a factor as well.

The dramatic decrease in transmissible infectious diseases leads to an opportunity to study the incidence of diagnoses that are assumed but not definitely proven to be caused by infections. While the base of circumstantial evidence to this effect is broad, the current analysis further adds to this. The relative reduction in assumed infection-related diagnoses was as large as the reduction for communicable infections. Our review of the literature found that similar reductions in pediatric healthcare utilization have been reported worldwide (Table 1). Besides general effects on the total volume of pediatric care, several studies reported on the effect of lockdown for individual diagnoses. For example, ED visits because of asthma decreased by 76–84% [30, 39], and Tacquechel et al. reported a 87% decrease in daily outpatient visits due to asthma and an 84% decrease in hospital visits (ED and inpatient) [49]. Shifts in the incidence of diagnoses in other specialties were also reported. For example, hospital admissions to a dermatology department in Poland reduced by 85% [50]. Finally, it is important to note that the pandemic and the accompanying lockdowns may also exhibit negative effects on mental health. For example, a 25% increase in diagnoses related to mental health and a 104% increase in admissions due to anorexia nervosa have been reported [51, 52]. On the other hand, a 27% reduction in mental health–related diagnoses as reason for ED visits was reported in Ireland [36]. The reduction in healthcare utilization was comparable to many other countries, despite the limited lockdown imposed in the Netherlands.

Our study has several limitations. First, since noncommunicable infections were a relatively small percentage of total diagnoses and as a result, the effect of lockdown on this category was difficult to estimate. Second, data on the level of ED acuity, length of hospital admission, and ICU admissions could have provided additional insights but were not available. Furthermore, due to different electronic management systems in each hospital, data exports were conducted individually for each study center. Although the requested data was specific, this may have led to small numbers of diagnoses that have been missed.

Strengths of this study include the multicenter approach, which makes this one of the most comprehensive analyses to date, allowed for the use of a mixed effects model and for the precise estimation of the average effects of lockdown in the Netherlands. Furthermore, the stratification of diagnoses in four categories gives a better overview of the specific effects of lockdown on the propensity of parents and children to go to the hospital during lockdowns. Future analyses may replicate the current findings but should also focus on the acuity of the clinical presentations and the proportion of delayed diagnoses or treatment.

## Conclusion

The COVID-19 pandemic and associated lockdowns caused a significant decrease in pediatric ED visits and hospital admissions in both the Netherlands as the rest of the world. While a large proportion of the reduction can be attributed to a decrease in transmissible infections not caused by SARS-CoV-2, our analyses show that avoidance of care could be a factor as well. Pediatricians should be aware of this possibility in the event of future lockdowns in their countries.

## Supplementary information

ESM 1 (PDF 714 kb)

## Data Availability

All data presented in this manuscript is available from the corresponding author upon reasonable request.

## Abbreviations

CI: Confidence interval
COVID-19: Coronavirus disease 2019
ED: Emergency department
PED: Pediatric emergency department
UTI: Urinary tract infection
