# Supplementary material for: The impact of lockdown on pediatric ED visits and hospital admissions during the COVID19 pandemic: a multicenter analysis and review of the literature
Source: Eur J Pediatr. 2021 Mar 15;180(7):2271–9. doi: 10.1007/s00431-021-04015-0 (PMC7959585; doi:10.1007/s00431-021-04015-0)
Supplement: Supplementary file 1 — (PDF 714 kb) [file 431_2021_4015_MOESM1_ESM.pdf]

Supplementary Table S1. List of diagnoses in each category

Group 1 (communicable infection)	Group 2 (infection-related)	Group 3 (noncommunicable infection)	Group 4 Non-infectious
Upper airway infection		Endocarditis	Gastro-intestinal disease*
Laryngitis subglottica	Asthma / Reactive airways	Vulvovaginitis	Neurological disease*
Mastoiditis/ethmoiditis	Cystic Fibrosis	UTI / Pyelonephritis	Nephrological disease*
Sinusitis	Myocarditis	Skeletal infections	Urological disease*
Whooping cough	Febrile convulsion	Granulopenic fever	Hematological disease*
Lower airway infection	Guillain-Barré		Malignant disease*
RSV	Glomerulonephritits	Cat scratch disease	Ocular disease*
Gastro-enteritis	Acute rheumatic fever	Lyme disease	Endocrine disease*
Hepatitis	Henoch Schonlein	Appendicitis	Cardiac disorder*
Meningitis/encephalitis	Kawasaki	Balanitis	Immune deficiencies
Ocular infection	Reactive arthritis	Epididymitis	JCA/JIA
Sepsis	Coxitis Fugax		Other autoimmune/ rheumatologic disease
Viral exanthema	ITP		Myalgia/artralgia
Lymphadenitis	Diabetes Mellitus		Hip dysplasia
Mononucleosis infectiosa	Viral-induced wheezing		Congenital disorder
Scarlet fever	Invagination		Syndrome
Viral infection NOS			Non-infectious exanthema
Varicella zoster infection			Metabolic disease
Other infectious disease			Laryngomalacia
Fever unknown origin			Aspiration
Laryngotrachobronchitis			BPD
Tuberculosis			Breath holding spells
Parasitic intestinal infection			Eating disorder
Skin infection			Excessive crying
Infection NOS			Psychiatric illness
Otitis media			Intoxication
Pericarditis			Coma
Stomatitis			Near-drowning
HIV			Burn
			Anaphylaxis / allergic reaction
			Failure-to-thrive
			Fatigue

Abbreviations: ITP: immune thrombocytopenic purpura RSV: respiratory syncytial virus, HIV: human immunodeficiency virus, UTI: urinary tract infection NOS: not otherwise specified, BPD: bronchopulmonary dysplasia. * Includes all diagnoses in this area unless specified in group 1-3.

Supplementary Text S2. Literature review search strategy

Source	Total	Unique hits after de-duplication
Embase.com	1194	1179
Medline ALL Ovid	1037	279
Web of Science Core Collection	513	166
CINAHL EBSCOhost	406	151
**Total**	**3150**	**1775**

Embase.com

('lockdown'/exp OR 'social distance'/exp OR 'social distancing'/exp OR 'social isolation'/de OR 'quarantine'/de OR 'coronavirus disease 2019'/de OR 'Severe acute respiratory syndrome coronavirus 2'/de OR ('pandemic'/de AND 'Coronavirus infection'/exp) OR (lockdown OR ((social) NEAR/3 (distan* OR isolation*)) OR quarantin* OR covid-19 OR covid19 OR sars-cov-2 OR sars-cov2 OR (Home NEAR/3 confine*) OR severe-acute-respiratory-syndrome-coronavirus-2 OR 2019-NCoV OR 2019-novel-coronavirus OR coronavirus-disease-2019 OR coronavirus-pandemic OR Novel-2019-coronavirus OR school-closure* OR daycare-closure* OR day-care-closure*):ab,ti) AND ('emergency ward'/exp OR 'hospital emergency service'/exp OR 'high dependency unit'/exp OR 'intensive care unit'/exp OR 'intermediate care unit'/exp OR 'nursing unit'/exp OR 'observation unit'/exp OR 'pediatric ward'/exp OR 'surgical ward'/exp OR 'hospital admission'/de OR 'emergency health service'/de OR 'emergency patient'/de OR (ward OR wards OR ((medium OR intensive OR paediatric OR pediatric OR low OR emergency) NEAR/3 (unit*)) OR ((emergenc*) NEAR/3 (department* OR room* OR unit* OR visit* OR service* OR setting)) OR admission* OR admitted* OR icu OR picu OR nicu OR er OR ed OR (hospital* NEAR/3 visit*)):ab,ti) AND (child/exp OR adolescent/exp OR adolescence/exp OR 'child behavior'/de OR 'child parent relation'/de OR pediatrics/exp OR childhood/exp OR 'child nutrition'/de OR 'infant nutrition'/exp OR 'child welfare'/de OR 'child abuse'/de OR 'child advocacy'/de OR 'child development'/de OR 'child growth'/de OR 'child health'/de OR 'child health care'/exp OR 'child care'/exp OR 'childhood disease'/exp OR 'child death'/de OR 'child psychiatry'/de OR 'child psychology'/de OR 'pediatric ward'/de OR 'pediatric hospital'/de OR 'pediatric anesthesia'/de OR 'pediatric intensive care unit'/de OR 'neonatal intensive care unit'/de OR 'prematurity'/de OR (adolescen* OR preadolescen* OR infan* OR newborn* OR (new NEXT/1 born*) OR baby OR babies OR neonat* OR prematur* OR pre-matur* OR child* OR kid OR kids OR toddler* OR teen* OR boy* OR girl* OR minors OR underag* OR (under NEXT/1 (age* OR aging OR ageing)) OR juvenil* OR youth* OR kindergar* OR puber* OR pubescen* OR prepubescen* OR prepubert* OR pediatric* OR paediatric* OR school* OR preschool* OR highschool* OR suckling* OR PICU OR NICU OR PICUs OR NICUs):ab,ti,kw) AND [2020]/py

Medline ALL Ovid

(Social Distance/ OR Social Isolation/ OR Quarantine/ OR coronavirus disease 2019/ OR Severe acute respiratory syndrome coronavirus 2/ OR (Pandemics/ AND Coronavirus Infections/) OR (lockdown OR ((social) ADJ3 (distan* OR isolation*)) OR quarantin* OR covid-19 OR covid19 OR sars-cov-2 OR sars-cov2 OR (Home ADJ3 confine*) OR severe-acute-respiratory-syndrome-coronavirus-2 OR 2019-NCoV OR 2019-novel-coronavirus OR coronavirus-disease-2019 OR coronavirus-pandemic OR Novel-2019-coronavirus OR school-closure* OR daycare-closure* OR day-care-closure*).ab,ti.) AND (Emergencies/ OR exp Emergency Service, Hospital/ OR Emergency Treatment/ OR exp Intensive Care Units/ OR Clinical Observation Units/ OR Patient Admission/ OR exp Emergency Medical Services/ OR (ward OR wards OR ((medium OR intensive OR paediatric OR pediatric OR low OR emergency) ADJ3 (unit*)) OR ((emergenc*) ADJ3 (department* OR room* OR unit* OR visit* OR service* OR setting)) OR admission* OR admitted* OR icu OR picu OR nicu OR er OR ed OR (hospital* ADJ3 visit*)).ab,ti.) AND (exp Child/ OR exp Infant/ OR exp Adolescent/ OR exp "Child Behavior"/ OR exp "Parent Child Relations"/ OR exp "Pediatrics"/ OR "Child Nutrition Sciences"/ OR "Infant nutritional physiological phenomena"/ OR exp "Child Welfare"/ OR "Child Development"/ OR exp "Child Health Services"/ OR exp "Child Care"/ OR "Child Rearing"/ OR exp "Child development Disorders, Pervasive"/ OR "Child Psychiatry"/ OR "Child Psychology"/ OR "Hospitals, Pediatric"/ OR exp "Intensive Care Units, Pediatric"/ OR (adolescen* OR preadolescen* OR infan* OR newborn* OR (new ADJ born*) OR baby OR babies OR neonat* OR prematur* OR pre-matur* OR child* OR kid OR kids

OR toddler* OR teen* OR boy* OR girl* OR minors OR underag* OR (under ADJ (age* OR aging OR ageing)) OR juvenil* OR youth* OR kindergar* OR puber* OR pubescen* OR prepubescen* OR prepubert* OR pediatric* OR paediatric* OR school* OR preschool* OR highschool* OR suckling* OR PICU OR NICU OR PICUs OR NICUs).ab,ti,kw.) AND 2020.yr.

Web of Science Core Collection

TS=(((lockdown OR ((social) NEAR/2 (distan* OR isolation*)) OR quarantin* OR covid-19 OR covid19 OR sars-cov-2 OR sars-cov2 OR (Home NEAR/2 confine*) OR severe-acute-respiratory-syndrome-coronavirus-2 OR 2019-NCoV OR 2019-novel-coronavirus OR coronavirus-disease-2019 OR coronavirus-pandemic OR Novel-2019-coronavirus OR school-closure* OR daycare-closure* OR day-care-closure*)) AND ((ward OR wards OR ((medium OR intensive OR paediatric OR pediatric OR low OR emergency) NEAR/2 (unit*)) OR ((emergenc*) NEAR/2 (department* OR room* OR unit* OR visit* OR service* OR setting)) OR admission* OR admitted* OR icu OR picu OR nicu OR er OR ed OR (hospital* NEAR/2 visit*))) AND ((adolescen* OR preadolescen* OR infan* OR newborn* OR (new NEAR/1 born*) OR baby OR babies OR neonat* OR prematur* OR pre-matur* OR child* OR kid OR kids OR toddler* OR teen* OR boy* OR girl* OR minors OR underag* OR (under NEAR/1 (age* OR aging OR ageing)) OR juvenil* OR youth* OR kindergar* OR puber* OR pubescen* OR prepubescen* OR prepubert* OR pediatric* OR paediatric* OR school* OR preschool* OR highschool* OR suckling* OR PICU OR NICU OR PICUs OR NICUs)))

CINAHL EBSCOhost

(MH Social Distancing OR MH Social Isolation OR MH Quarantine OR MH COVID-19 OR (MH Disease Outbreaks AND MH Coronavirus Infections) OR TI(lockdown OR ((social) N2 (distan* OR isolation*)) OR quarantin* OR covid-19 OR covid19 OR sars-cov-2 OR sars-cov2 OR (Home N2 confine*) OR severe-acute-respiratory-syndrome-coronavirus-2 OR 2019-NCoV OR 2019-novel-coronavirus OR coronavirus-disease-2019 OR coronavirus-pandemic OR Novel-2019-coronavirus OR school-closure* OR daycare-closure* OR day-care-closure*) OR AB(lockdown OR ((social) N2 (distan* OR isolation*)) OR quarantin* OR covid-19 OR covid19 OR sars-cov-2 OR sars-cov2 OR (Home N2 confine*) OR severe-acute-respiratory-syndrome-coronavirus-2 OR 2019-NCoV OR 2019-novel-coronavirus OR coronavirus-disease-2019 OR coronavirus-pandemic OR Novel-2019-coronavirus OR school-closure* OR daycare-closure* OR day-care-closure*)) AND (MH Emergencies OR MH Emergency Service+ OR MH Emergency Treatment OR MH Intensive Care Units+ OR MH Observation Units OR MH Patient Admission OR MH Emergency Medical Services OR TI(ward OR wards OR ((medium OR intensive OR paediatric OR pediatric OR low OR emergency) N2 (unit*)) OR ((emergenc*) N2 (department* OR room* OR unit* OR visit* OR service* OR setting)) OR admission* OR admitted* OR icu OR picu OR nicu OR er OR ed OR (hospital* N2 visit*)) OR AB(ward OR wards OR ((medium OR intensive OR paediatric OR pediatric OR low OR emergency) N2 (unit*)) OR ((emergenc*) N2 (department* OR room* OR unit* OR visit* OR service* OR setting)) OR admission* OR admitted* OR icu OR picu OR nicu OR er OR ed OR (hospital* N2 visit*))) AND (MH Child+ OR MH Infant+ OR MH Adolescent+ OR MH "Child Behavior+" OR MH "Pediatrics+" OR MH "Child Health Services+" OR MH "Child Care+" OR MH "Child Rearing" OR MH "Hospitals, Pediatric" OR MH "Intensive Care Units, Pediatric+" OR TI(adolescen* OR preadolescen* OR infan* OR newborn* OR (new N1 born*) OR baby OR babies OR neonat* OR prematur* OR pre-matur* OR child* OR kid OR kids OR toddler* OR teen* OR boy* OR girl* OR minors OR underag* OR (under N1 (age* OR aging OR ageing)) OR juvenil* OR youth* OR kindergar* OR puber* OR pubescen* OR prepubescen* OR prepubert* OR pediatric* OR paediatric* OR school* OR preschool* OR highschool* OR suckling* OR PICU OR NICU OR PICUs OR NICUs) OR AB(adolescen* OR preadolescen* OR infan* OR newborn* OR (new N1 born*) OR baby OR babies OR neonat* OR prematur* OR pre-matur* OR child* OR kid OR kids OR toddler* OR teen* OR boy* OR girl* OR minors OR underag* OR (under N1 (age* OR aging OR ageing)) OR juvenil* OR youth* OR kindergar* OR puber* OR pubescen* OR prepubescen* OR prepubert* OR pediatric* OR paediatric* OR school* OR preschool* OR highschool* OR suckling* OR PICU OR NICU OR PICUs OR NICUs))

Supplementary Figure S3. Hospital admission:ED visit ratio

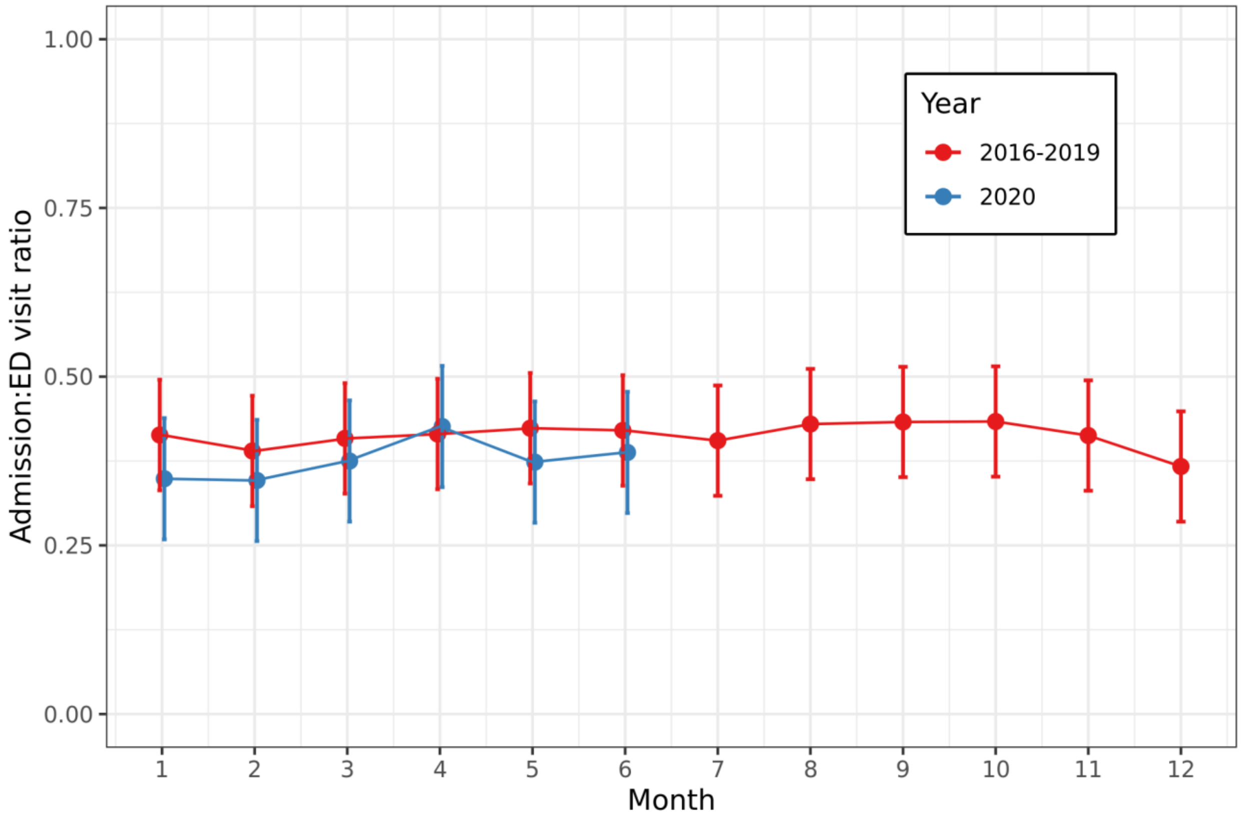

Estimated mean (95% CI) Admission:ED visit ratio per month for the 8 study centers for 2016-2019 (red) and 2020 (blue).

Supplementary Table S4. Estimated effects per month per category

All ED visits			
**Month**	**Difference (%)**	**95% CI**	**p-value***
**January**	-1.67	-9.96 – 6.62	1.000
**February**	-9.17	-17.46 – -0.88	0.541
**March**	-36.26	-44.55 – -27.98	**<0.001**
**April**	-59.31	-67.60 – -51.02	**<0.001**
**May**	-47.79	-56.08 – -39.50	**<0.001**
**June**	-40.60	-48.89 – -32.31	**<0.001**

ED visits group 1 (communicable infections)			
**Month**	**Difference (%)**	**95% CI**	**p-value***
**January**	-2.45	-14.51 – 9.61	1.000
**February**	-18.66	-30.72 – -6.60	**0.044**
**March**	-39.98	-52.04 – -27.92	**<0.001**
**April**	-75.94	-88.00 – -63.88	**<0.001**
**May**	-67.35	-79.41 – -55.29	**<0.001**
**June**	-62.85	-74.91 – -50.79	**<0.001**

ED visits group 2 (infection-related)			
**Month**	**Difference (%)**	**95% CI**	**p-value***
**January**	-0 .62	-18.16 – 16.91	1.000
**February**	-14.54	-32.08 – 3.00	1.000
**March**	-47.06	-64.59 – -29.52	**<0.001**
**April**	-71.33	-88.87 – -53.80	**<0.001**
**May**	-67.46	-85.00 – -49.92	**<0.001**
**June**	-38.76	-56.30 – -21.22	**<0.001**

ED visits group 3 (non-communicable infections)			
**Month**	**Difference (%)**	**95% CI**	**p-value***
**January**	-3.70	-31.99 – 24.59	1.000
**February**	20.39	-7.90 – 48.68	1.000
**March**	-5.40	-33 .69 – 22.88	1.000
**April**	-7.73	-36.02 – 20.55	1.000
**May**	-29.17	-57.46 – -0.89	0.778
**June**	-14.60	-42.89 – 13.69	1.000

ED visits group 4 (non-infectious disease)			
**Month**	**Difference (%)**	**95% CI**	**p-value***
**January**	0.99	-9.18 – 11.16	1.000
**February**	5.78	-4.39 – 15.94	1.000
**March**	-28.56	-38.72 – -18.39	**<0.001**
**April**	-36.16	-46.33 – -25.99	**<0.001**
**May**	-23.51	-33.68 – -13.35	**<0.001**
**June**	-24.54	-34.70 – -14.37	**<0.001**

All admissions			
**Month**	**Difference (%)**	**95% CI**	**p-value***
**January**	-16.50	-26.47 – -6.53	**0.021**
**February**	-17.28	-27.25 – -7.31	**0.012**
**March**	-40.94	-50.91 – -30.97	**<0.001**
**April**	-56.80	-66.77 – -46.83	**<0.001**
**May**	-53.96	-63.93 – -43.99	**<0.001**
**June**	-43.68	-53.65 – -33.72	**<0.001**

Admissions group 1 (communicable infections)			
**Month**	**Difference (%)**	**95% CI**	**p-value***
**January**	-13.94	-28.21 – 0.33	1.000
**February**	-23.81	-38.08 – --9.54	**0.019**
**March**	-46.59	-60.86 – -32.32	**<0.001**
**April**	-77.29	-91.56 – -63.02	**<0.001**
**May**	-72.41	-86.68 – -58.14	**<0.001**
**June**	-68.55	-82.82 – -54.28	**<0.001**

Admissions group 2 (infection-related)			
**Month**	**Difference (%)**	**95% CI**	**p-value***
**January**	-11.85	-36.86 – 13.17	1.000
**February**	-14.19	-39.21 – 10.82	1.000
**March**	-43.51	-68.53 – -18.49	**0.012**
**April**	-78.28	-103.30 – -53.27	**<0.001**
**May**	-78.42	-103.43 – -53.40	**<0.001**
**June**	-39.61	-64.63 – -14.60	**<0.034**

Admissions group 3 (non-communicable infections)			
**Month**	**Difference (%)**	**95% CI**	**p-value***
**January**	10.75	-39.96 – 61.47	1.000
**February**	14.01	-36.71 – 64.72	1.000
**March**	-28.07	-78.78 – 22.65	1.000
**April**	9.57	-41.15 – 60.28	1.000
**May**	-26.10	-76.82 – 24.61	1.000
**June**	-18.55	-69.26 – 32.17	1.000

Admissions group 4 (non-infectious disease)			
**Month**	**Difference (%)**	**95% CI**	**p-value***
**January**	-20.36	-33.37 – -7.34	**0.039**
**February**	-9.23	-22.25 – 3.78	1.000
**March**	-33.35	-46.37 – -20.34	**<0.001**
**April**	-30.69	-43.71 – -17.68	**<0.001**
**May**	-30.37	-43.39 – -17.36	**<0.001**
**June**	-24.21	-37.22 – -11.19	**0.005**

Supplementary Figure S5. Incidence of selected diagnoses

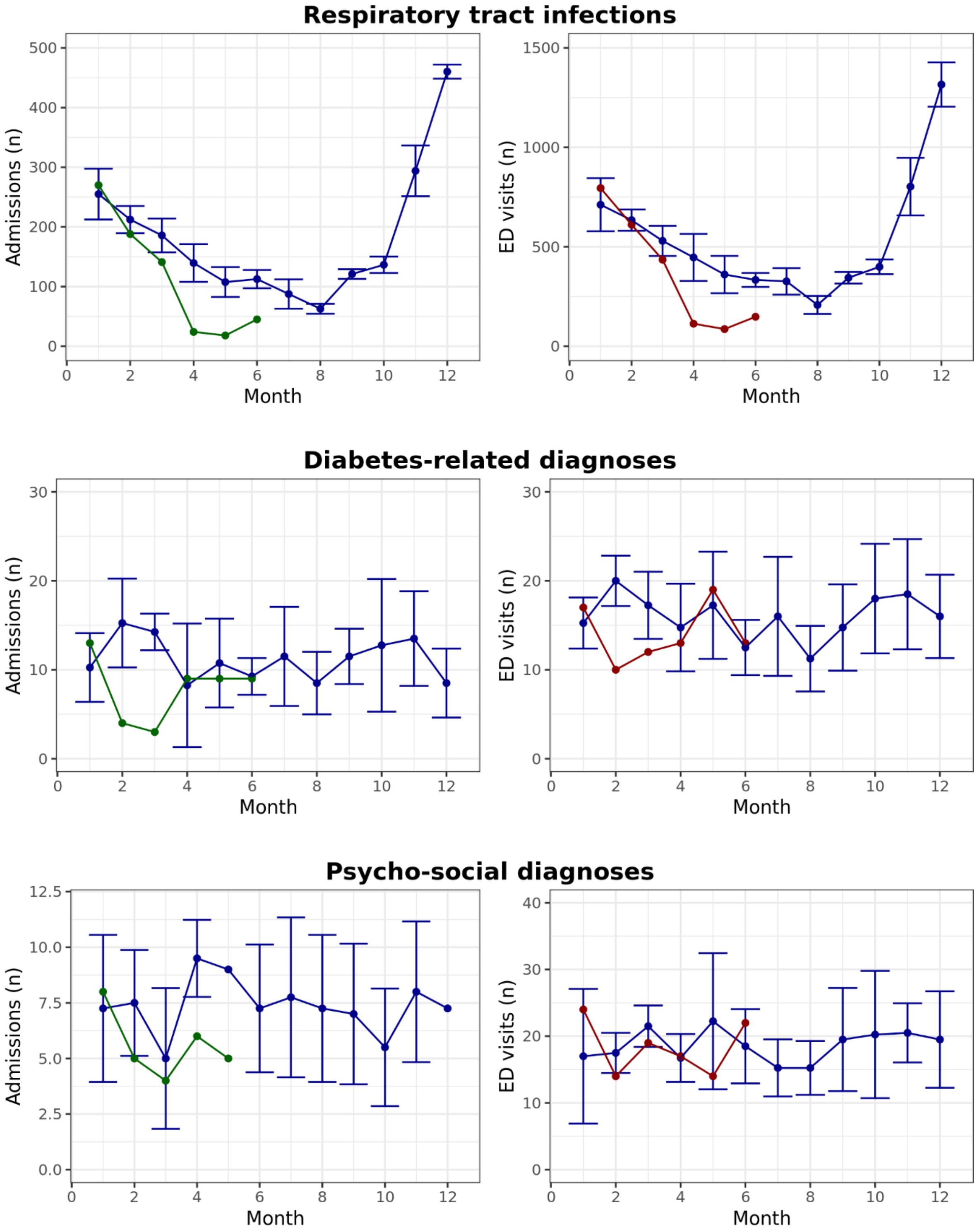

Supplementary Figure S6 – Flowchart literature review

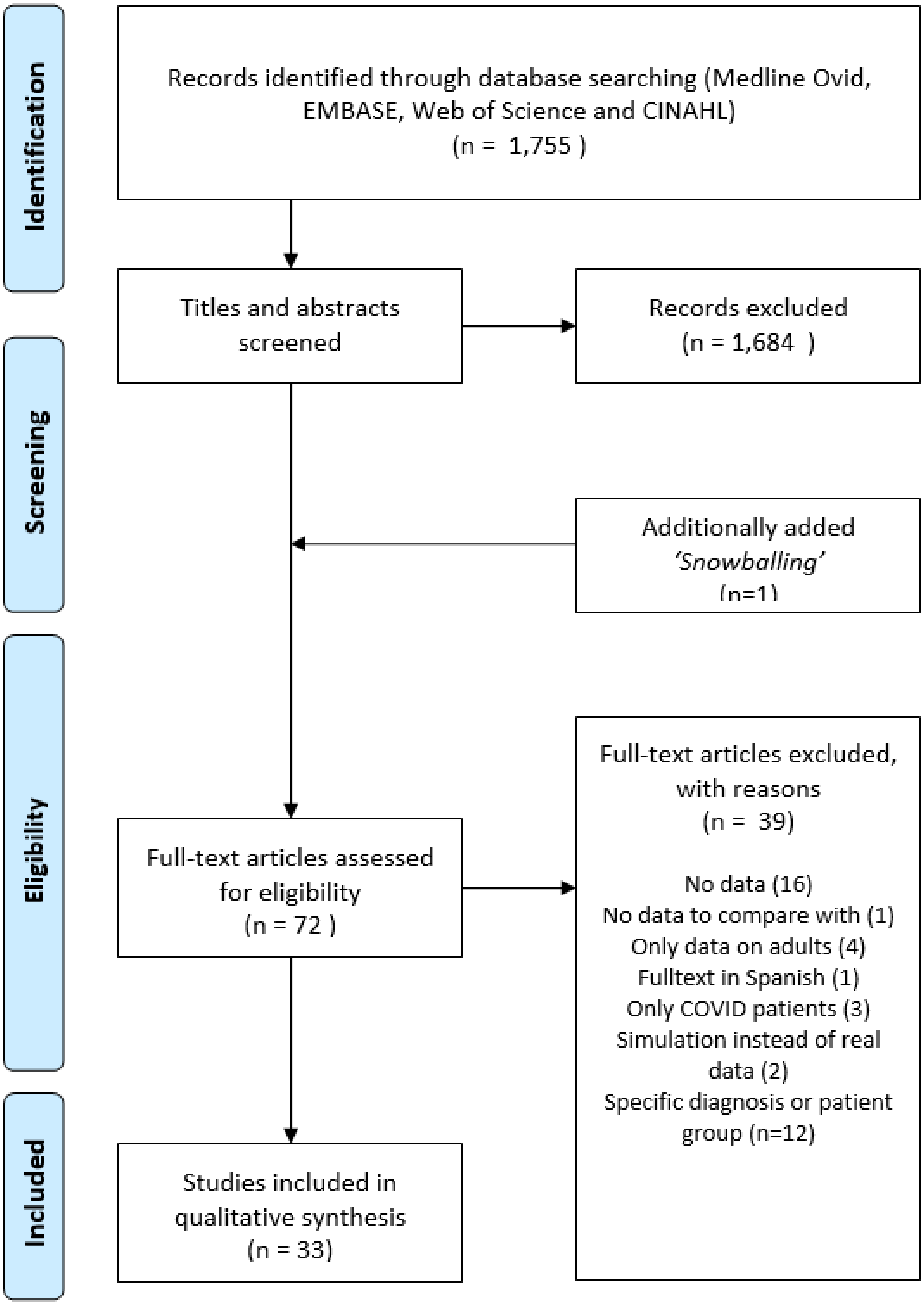

Supplementary Figure S7 – Correlation reduction in ED visits and maximum lockdown stringency

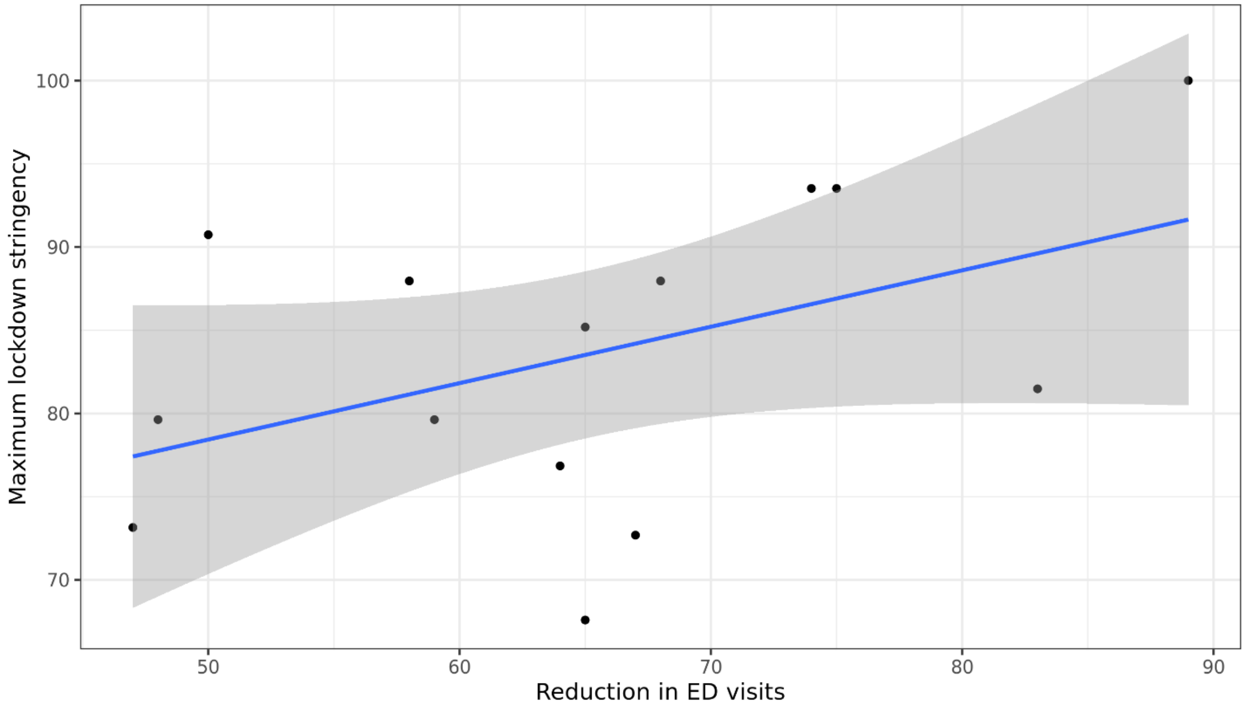

Correlation between the reduction in ED visits per country and the maximum lockdown stringency. Each dot represents a country. Blue line and shaded area represents the linear regression line and 95% confidence interval.
